# Comparative assessment of anti-cancer drugs against NUDT15 variants to prevent leucopenia side effect in leukemia patients

**DOI:** 10.1186/s43141-023-00538-1

**Published:** 2023-08-09

**Authors:** Janakiraman V., Sudhan M., Khalaf F. Alsharif, Ibrahim F. Halawani, Shiek S. S. J. Ahmed, Shankargouda Patil

**Affiliations:** 1 Drug Discovery and Multi-Omics Laboratory, Faculty of Allied Health Sciences, Chettinad Hospital and Research Institute, Chettinad Academy of Research and Education, Kelambakkam-603103, Tamil Nadu, India; 2 Department of Clinical Laboratories Sciences, College of Applied Medical Sciences, Taif University, P.O. Box 11099, 21944 Taif, Saudi Arabia; 3 College of Dental Medicine, Roseman University of Health Sciences, South Jordan, UT USA

**Keywords:** NUDT15, Leukemia, Drugs, Treatment, Docking, Simulation, Azathioprine, Mercpatopurine, Thioguanine

## Abstract

**Background:**

Human nucleotide triphosphate diphosphatase (NUDT15) is one of the essential proteins involved in the hydrolysis of anti-cancer drugs against leukemia. Polymorphisms in NUDT15 significantly affect the hydrolysis activity that leads to side effects, including leucopenia. Drugs having a better affinity with NUDT15 protein and contributing stable conformation may benefit patients from leucopenia. Most frequent NUDT15 polymorphisms causing structure variability and their association with leukemia were screened. The selected protein variants and anti-cancer drug structures were collected. Further, molecular docking was performed between drugs and NUDT15 variants along with the wild-type. Finally, molecular dynamics were executed for 100 ns to understand the stability of the protein with the anti-cancer drug based on molecular trajectories.

**Results:**

Three-dimensional structures of NUDT15 wild, the most frequent variants (Val18Ile, Arg139Cys, and Arg139), and the anti-cancer drugs (azathioprine, mercaptopurine, and thioguanine) were selected and retrieved from structure databases. On molecular docking the binding energies of anti-cancer drugs against NUDT15 structures ranged from − 5.0 to − 5.9 kcal/mol. Among them, azathioprine showed the highest affinities (− 7.3 kcal/mol) for the wild and variant structures. Additionally, the molecular dynamics suggest all analyzed NUDT15 were stable with azathioprine based on the dynamic trajectories.

**Conclusion:**

Our results suggest azathioprine could be the preferable anti-cancer drug for the population with NUDT15 variants that could effectively be hydrolyzed as evidenced by molecular docking and dynamic simulation.

**Supplementary Information:**

The online version contains supplementary material available at 10.1186/s43141-023-00538-1.

## Background

Leukemia is a type of blood cancer that originates during the process of hematopoiesis in the lymphatic system and bone marrow [1]. Several genetic and environmental factors are linked with leukemia that causes the rapid proliferation of abnormal blood cells [2]. Globally compared to adults, children are more prone to acute lymphoblastic leukemia [3]. The National Cancer Institute reported 14.1 per 100,000 new leukemia cases in 2015–2019 (https://www.cancer.gov/types/leukemia) and the mortality rate was 6.0 per 100,000 in both men and women (https://www.cancer.gov/types/leukemia). Leukemia alters the structural and molecular properties of leukocytes. Few studies have reported the polymorphisms in NUDT15, IL-10, FOXP3, TLR, NLR, and RLR genes were associated with leukemia. Among them, nudix hydrolase 15 (NUDT15) is directly associated with leukemia as well as with cancer drug metabolism [4–9].

Human NUDT15 is located at chromosome 13 positioned in long arm 14.2 consisting of 164 amino acids [10]. The NUDT15 plays an important role in the hydrolysis of nucleotide triphosphates including dTTP, dCTP, and dGTP [11]. Particularly, the low enzymatic activity of the NUDT15 increases thiopurine accumulation, which causes severe toxic effects, including DNA breakage and apoptosis [12, 13]. Previous studies report genetic polymorphisms in NUDT15 to affect the metabolism of thiopurine drugs (azathioprine, mercaptopurine, and thioguanine) that are associated with leukemia treatment [14]. Interestingly, patients with inflammatory bowel disease with NUDT15 polymorphisms are susceptible to thiopurine toxicity and they required the minimum dose of drug reported by a Korean study [15]. Similarly in childhood acute lymphoblastic leukemia, NUDT15 polymorphisms were linked with thiopurine related myelosuppression [16]. Thiopurines were clinically used to treat different kinds of cancer, NUDT15 playing a major role in the metabolism of thiopurines [17]. Due to genetic polymorphism, the function of the NUDT15 may altered and would affect drug metabolism thereby lead to several health complications. Therefore, patients with NUDT15 polymorphisms required the critical recommendation of thiopurine drugs to avoid leucopenia complications in leukemia patients [17, 18].

In this study, we critically evaluate the structural behavior of wild and three more frequently observed polymorphic variants of NUDT15 protein by implementing a variety of computational approaches*.* Particularly, the binding affinity of thiopurine-derived drugs to the NUDT15 variants was assessed by molecular docking and dynamic simulation. Our analyses provide therapeutic insights towards the most suitable thiopurine drugs appropriate to NUDT15 polymorphism that elucidate the effective treatment strategy to avoid leucopenia complications in patients with leukemia.

## Methods

### Retrieval of NUDT15 amino acid sequences and variants

The protein sequence of the human NUDT15 was obtained from the Uniprot database using accession number Q9NV35 [19]. Further, the variants contributing changes in amino acid (missense) were selected using the Ensembl variant table [20]. Then, Genome Aggregation Database (GnomAD) v2.1.1 database was utilized to determine the frequencies of the selected variants across various populations [21]. The most prevalent variants across the population (East Asian, South Asian, European (Finnish), European (Non-Finnish), Latino admixed American, African/African American, Ashkenazi Jewish, and others) were selected as the candidate variants for further analysis.

### Protein–ligand preparation

Most recommended anti-cancer thiopurine-derived drugs (1) azathioprine (CID: 2265), (2) mercaptopurine (CID: 667490), and (3) thioguanine (CID: 2723601) against leukemia were selected and their structures were downloaded in structure data file (sdf) format from the PubChem database [22]. The collected structures were optimized by applying an mmff94 force field and converted into an appropriate format for molecular docking [23]. Likewise, the three-dimensional structure of NUDT15 wild-type (5LPG) and the polymorphic variants Val18Ile (7B64), Arg139Cys (7B65), and Arg139His (7B66) were downloaded from the protein data bank (PDB) [24]. From the collected protein structure, the water molecules and heteroatoms were removed and energy was minimized with the mmff94 force field. Finally, both protein (*n* = 4; 1 wild-type + 3 polymorphic variants) and drugs (*n* = 3; azathioprine, mercaptopurine, and thioguanine) were processed for molecular docking.

### Mutational analysis of NUDT15 variants

Simultaneously, the three-dimensional structure of NUDT15 variants (Val18Ile, Arg139Cys, and Arg139His) were subjected to stability analysis using MAESTROweb [25], INPS-3D [26], CUPSAT [27], PremPS [28], and SDM2 [29]. Each tool predicts relative binding free energy (ΔΔG) using different algorithms to determine the structural stability. The obtained ΔΔG value was negative; it indicates mutation destabilizes the protein structure, whereas if ΔΔG value was positive it denotes a stable protein structure.

### Molecular docking analysis

Molecular docking was performed to identify the binding affinity of the protein-drug complex. Here, PyRx version 0.8 was used to perform protein-drug docking [30]. The Grid box was generated for the protein molecule using Pyrex Auto Grid. The optimum value of root mean square deviation (RMSD) was set < 1.0 Å, which was considered the most favorable binding interaction. Finally, the drug (azathioprine, mercaptopurine, and thioguanine) with the highest affinity towards each protein (wild-type, Val18Ile, Arg139Cys, and Arg139His) was selected for visualization using Discovery Studio V21.1.

### Molecular dynamics simulation

Molecular dynamics (MD) simulation using GROMACS 2021 was employed for each protein-drug complex [31]. The CHARMM-all atoms force field was applied and placed in an orthorhombic periodic boundary box with the dimension of 1 Å. The salt concentration was maintained at 0.150 M. The system was solvated using TIP3P water and neutralized by adding sodium ions. Further, 50,000 steps of the steepest descent approach were utilized for energy minimization. The temperature of the system is maintained at 300 K with a constant pressure of 1 bar by while structure equilibrium NVT (number of particles, volume, and temperature) and NPT (number of particles, pressure, temperature). The MD simulation was performed for 100 ns. Linear Constraint Solver (LCS) approach was engaged to constrain the system’s hydrogen bonds and Particle Mesh Ewald (PME) was used to calculate long-range electrostatic interaction. Further, the MD trajectories such as root mean square deviation (RMSD), root mean square fluctuation (RMSF), radius of gyration (RG), solvent accessible surface area (SASA), and hydrogen bond (HB) were analyzed and interpreted.

## Results

### Dataset collection and NUDT15 stability assessment

According to the Ensembl variant table, 83 missense variants were found localized in NUDT15. Based on the polymorphic frequency top three polymorphisms (Val18Ile, Arg139Cys, and Arg139His) were identified that are over-represented across various populations (Table 1). Further, the stability of the NUDT15 protein influenced by amino acid substitutions was determined using stability prediction tools. Analysis showed all three variants derived protein structures unanimously have destabilizing effects (Table 2).

**Table 1:** Population frequency analysis of the variants across various populations

SNP	Val18Ile	Arg139Cys	Arg139His
dbSNP ID	rs186364861	rs116855232	rs147390019
East Asian	0.01138	0.1048	0.001112
South Asian	0.0004502	0.06661	0.00003288
European (non-Finnish)	0.000009149	0.00351	0.00002329
European (Finnish)	0	0.02284	0
African/African American	0	0.001007	0.0002414
Latino admixed American	0	0.05959	0.01793
Ashkenazi Jewish	0.0001051	0.00377	0
Others	0.0004658	0.02065	0.00195

**Table 2:** Stability analysis of the NUDT15 variants (Val18Ile Arg139Cys, and Arg139His)

Tools	Val18Ile	Arg139Cys	Arg139His
Maestroweb	Stabilizing	Stabilizing	Stabilizing
CUPSAT	Destabilizing	Stabilizing	Destabilizing
SDM2	Destabilizing	Destabilizing	Stabilizing
INPS-3D	Destabilizing	Destabilizing	Destabilizing
PremPS	Stabilizing	Stabilizing	Stabilizing

### Molecular docking analysis

**Fig. 1:**
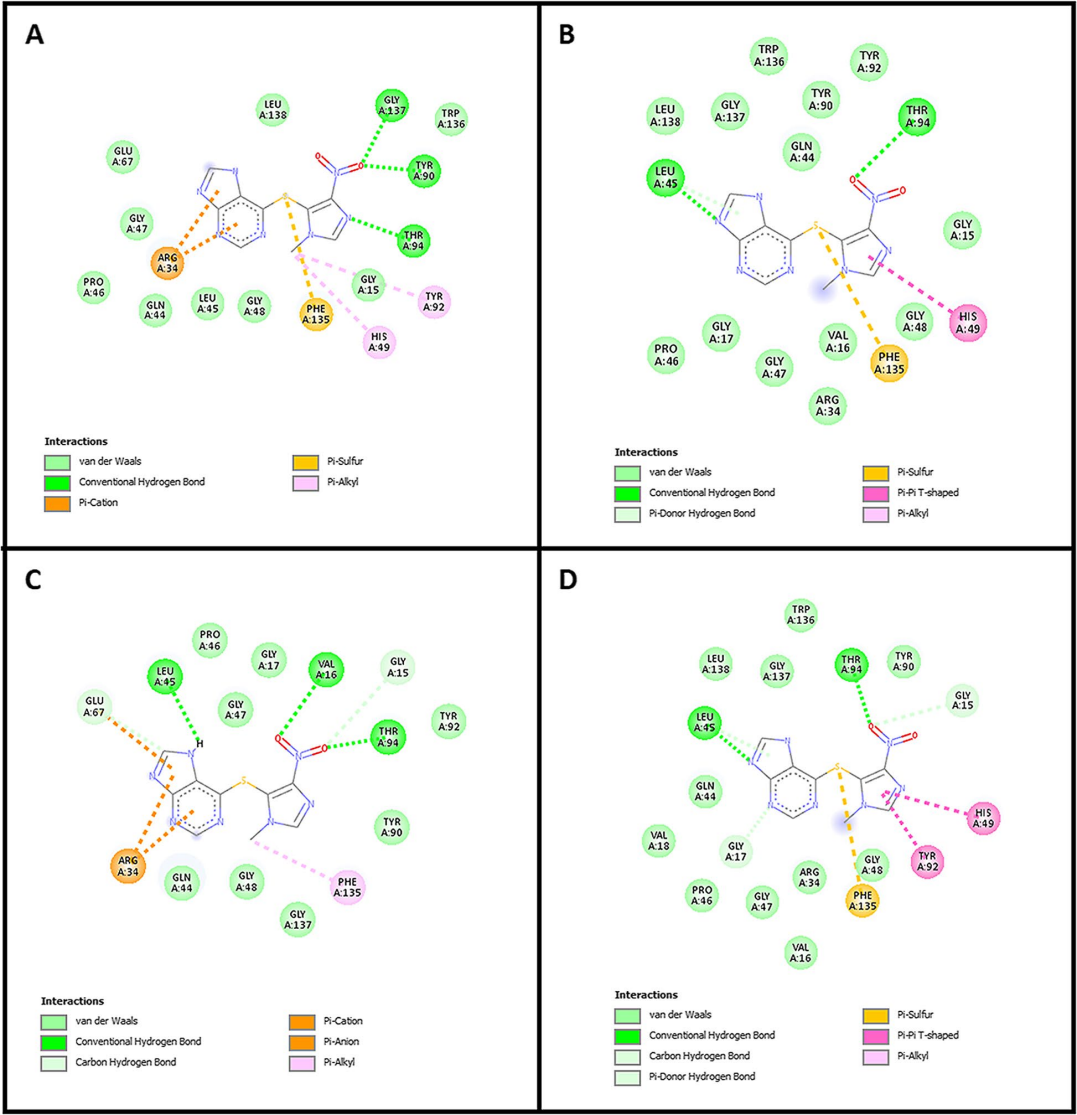
The interaction of azathioprine with NUDT15 wild-type with its variants (Val18Ile, Arg139Cys, and Arg139His). **A** Interaction pattern of azathioprine with wild-type. **B** Binding interaction of azathioprine with Val18Ile. **C** Azathioprine interaction with Arg139Cys. **D** Molecular interaction of Arg139His and azathioprine

Next, the docking analysis revealed the binding energy of azathioprine, mercaptopurine, and thioguanine to the NUDT15 wild-type and its variants (Val18Ile, Arg139Cys, and Arg139His). The binding energy of azathioprine, mercaptopurine, and thioguanine to the wild-type was represented in Table 3. Notably, azathioprine showed the highest affinity with a least binding energy of − 8.2 kcal/mol against Arg139Cys variant structure that interacts with GLU67, LEU45, ARG34, PHE135, THR94, GLY15, and VAL16 (Fig. 1).

**Table 3:** The binding energy of three thiopurine drugs with NUDT15 wild-type and its variants

NUDT15 Proteins	Thiopurine drugs		
	Azathioprine (kcal/mol)	Mercaptopurine (kcal/mol)	Thioguanine (kcal/mol)
Wild-type	− 7.6	− 5.6	− 6
Val18Ile	− 7.3	− 5.3	− 5.5
Arg139Cys	− 8.2	− 5.4	− 5.9
Arg139His	− 7.3	− 5.3	− 5.9

### Molecular dynamic simulation

**Fig. 2:**
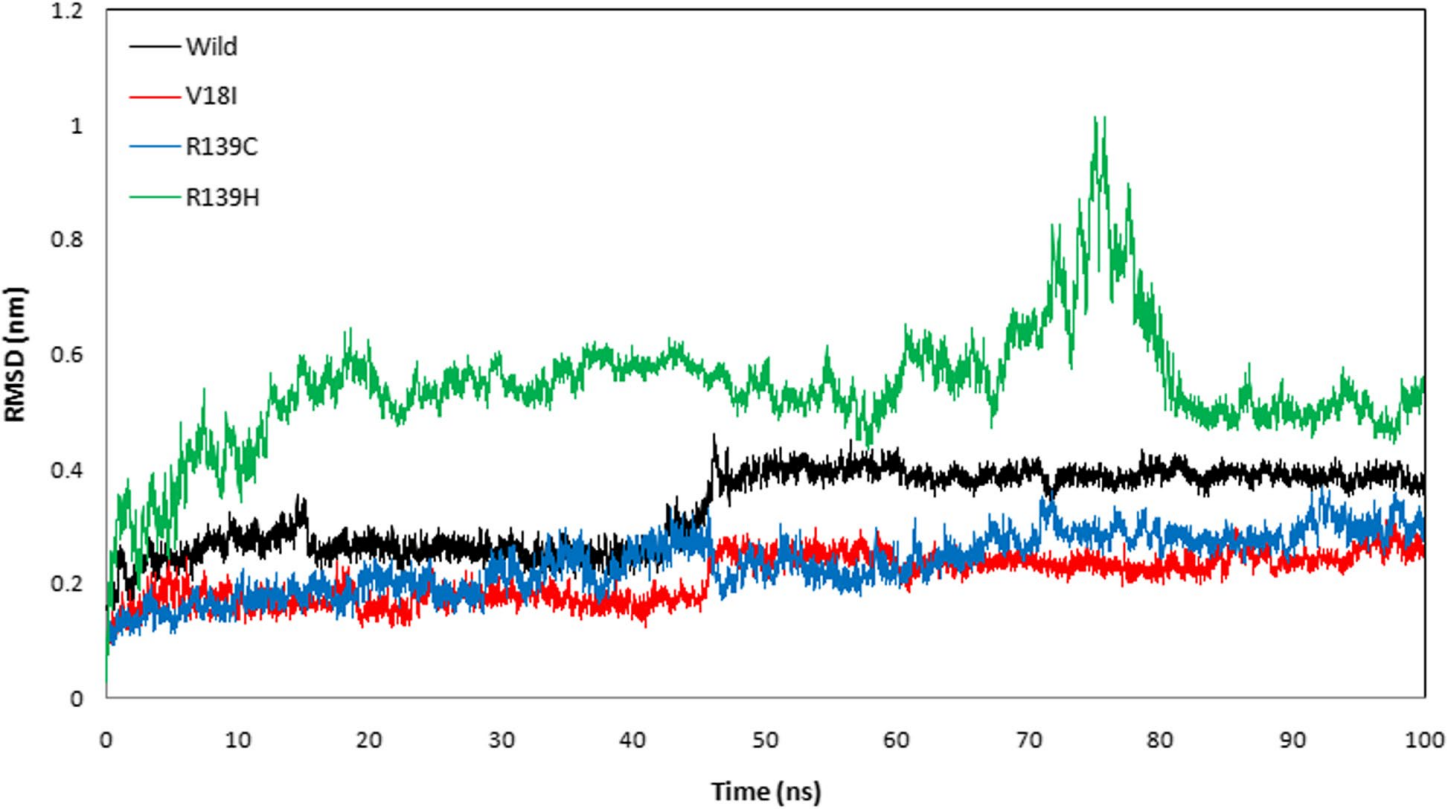
The root mean square deviation analysis of azathioprine with NUDT15 wild-type and its variants (Val18Ile, Arg139Cys, and Arg139His). The black color indicates the wild-type, while red color indicates Val18Ile. Likewise, blue color represents Arg139Cys and green color illustrates Arg139His

**Fig. 3:**
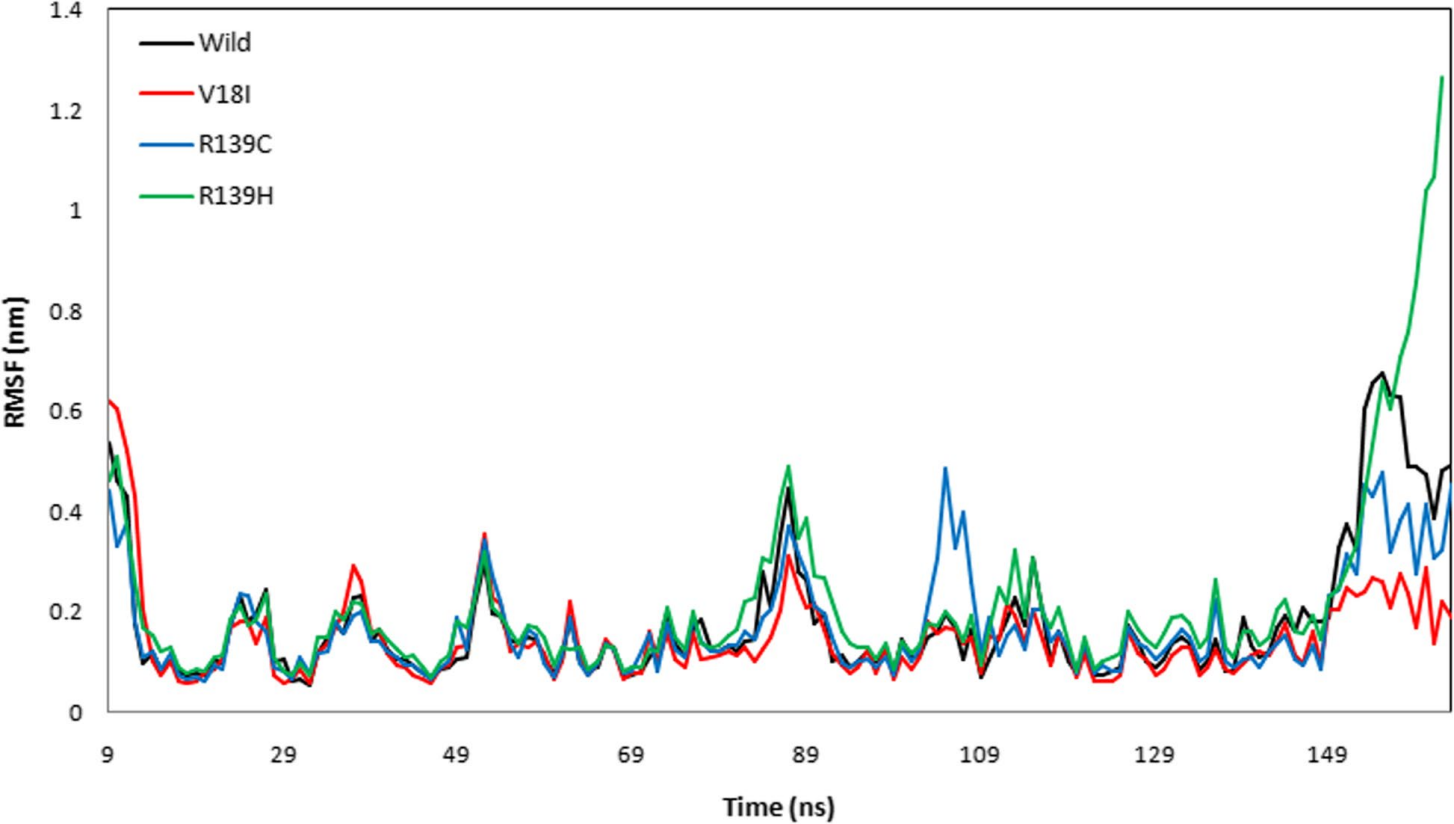
The protein flexibility of azathioprine with NUDT15 wild-type and its variants by root mean square fluctuation analysis. The black, red, blue, and green color indicates wild-type, Val18Ile, Arg139Cys, and Arg139His

**Fig. 4:**
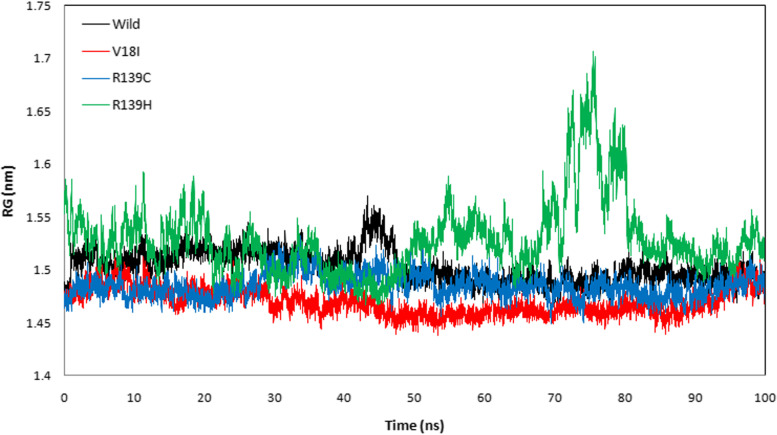
The radius of gyration of azathioprine with NUDT15 wild-type and its variants during 100 ns simulation. The NUDT15 wild-type, Val18Ile, Arg139Cys, and Arg139His were represented by black, red, blue, and green colors

**Fig. 5:**
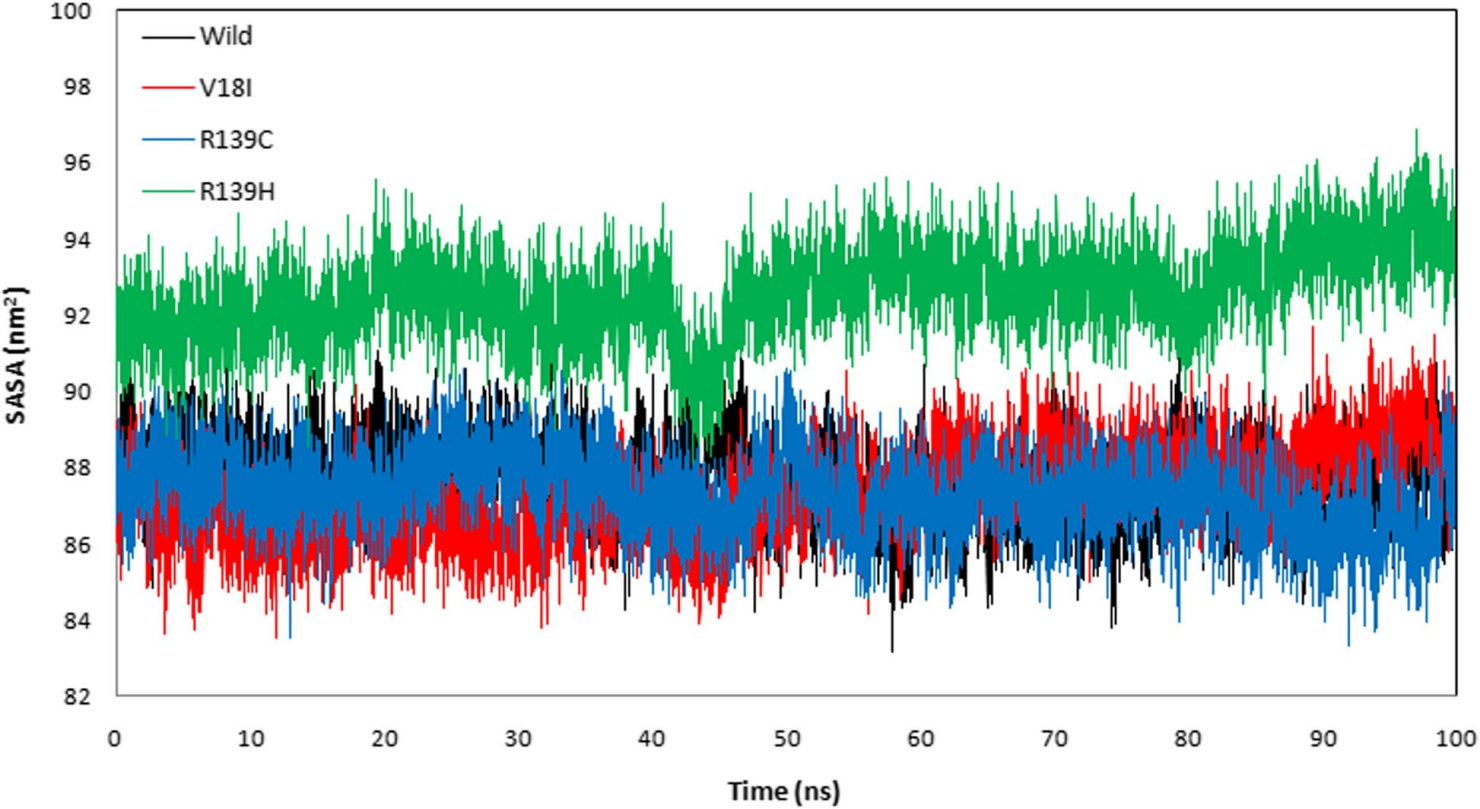
The surface area ofNUDT15 wild-type and its variants with azathioprine by SASA analysis. The black and red color illustrates NUDT15 wild-type and Val18Ile, while blue and green color shows NUDT15 Arg139Cys, and Arg139His

**Fig. 6:**
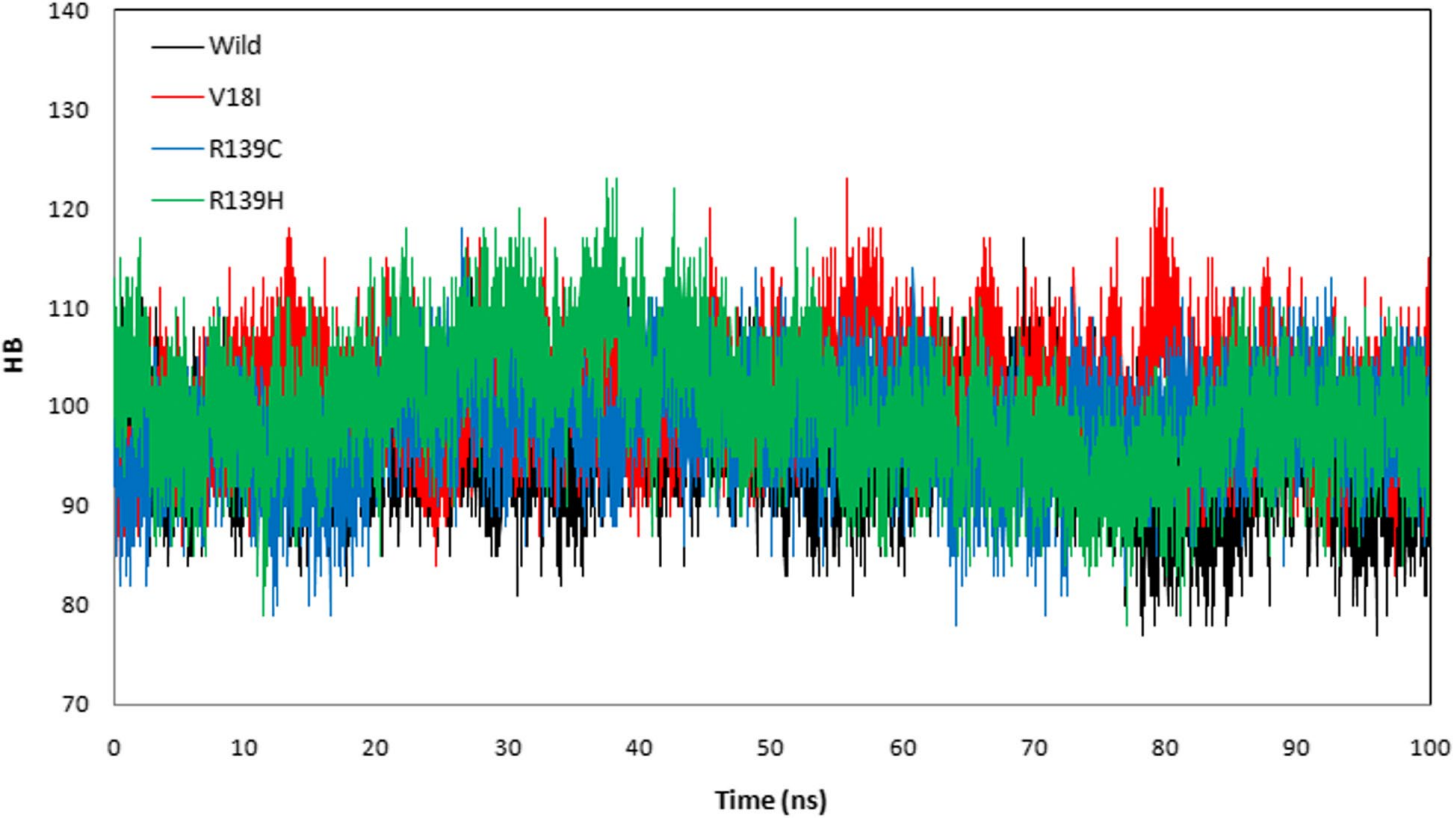
The azathioprine with NUDT15 wild-type and its variants hydrogen bond interaction. The NUDT15 wild-type (black), Val18Ile (red), Arg139Cys (blue), and Arg139His (green) shows the number of hydrogen bonds formed during 100 ns simulation with azathioprine

The protein trajectory was assessed based on RMSD, RMSF, RG, SASA, and HB to determine structural characteristics of NUDT15 wild-type and the variants (Val18Ile, Arg139Cys, and Arg139His) with the three anti-cancer drugs (Table 4). Our MD analysis with azathioprine showed the average RMSD was ranged between 0.210 and 0.541 nm and average RMSF was ranged between 0.149 and 0.236 nm for wild-type, Val18Ile, Arg139Cys, and Arg139His (Table 4). Likewise, the average RG ranged between 1.470 and 1.529 nm and the average SASA was between 87.296 and 92.583 nm^2^ (Table 4). Finally, the average HB ranged between 95. 490 – 101.159 for wild-type, Val18Ile, Arg139Cys, and Arg139His with azathioprine, respectively (Table 4). The pictorial representation of RMSD, RMSF, RG, SASA, and HB of azathioprine with NUDT15 wild-type, Val18Ile, Arg139Cys, and Arg139His with azathioprine were shown in Figs. 2, 3, 4, 5, and 6.

**Table 4:** The average RMSF, RG, SASA, and HB of the wild-type and its variants with azathioprine

Azathioprine (average protein trajectories)				
	Wild-type	Val18Ile	Arg139Cys	Arg139His
RMSD (nm)	0.331	0.21	0.236	0.541
RMSF (nm)	0.184	0.149	0.172	0.236
RG (nm)	1.502	1.47	1.485	1.529
SASA (nm^2^)	87.702	87.296	87.325	92.583
HB	95.49	101.159	97.887	99.768

*RMSD* Root mean square deviation, *RMSF* Root mean square fluctuation, *RG* Radius of gyration, *SASA* Solvent accessible surface area, *HB* Hydrogen bond

Alternatively, the average RMSD with mercaptopurine ranged between 0.205 and 0.494 nm for wild-type, Val18Ile, Arg139Cys, and Arg139His (Table 5). Likewise, the average RMSF ranged between 0.143 and 0.205 nm and the average RG was between 1.453 and 1.501 nm. The average SASA was between 86.985 and 91.354 nm^2^. Finally, the average HB ranged between 96.639 and 101.669 for wild-type, Val18Ile, Arg139Cys, and Arg139His with mercaptopurine (Table 5). Similarly, the average RMSD with thioguanine ranged between 0.314 and 0.446 nm, and the RMSF average ranged between 0.172 and 0.236 nm for wild-type, Val18Ile, Arg139Cys, and Arg139His. Likewise, the average RG ranged between 1.491 and 1.574 nm and the average SASA was between 88.281 and 92.321 nm^2^. Finally, the average HB ranged between 93.392 and 98.071 for wild-type, Val18Ile, Arg139Cys, and Arg139His with thioguanine (Table 6). The simulation figures of mercaptopurine and thioguanine with NUDT15 wild-type and its variants were represented in the supplementary file (Figures S1, S2, S3, S4, S5, S6, S7, S8, S9 and S10).

**Table 5:** The average simulation trajectories of the wild-type and its variants with mercaptopurine

Mercaptopurine (average protein trajectories)				
	Wild-type	Val18Ile	Arg139Cys	Arg139His
RMSD (nm)	0.211	0.369	0.205	0.494
RMSF (nm)	0.16	0.201	0.143	0.205
RG (nm)	1.49	1.498	1.453	1.501
SASA (nm^2^)	87.642	88.526	86.985	91.354
HB	101.669	96.639	101.522	98.589

*RMSD* Root mean square deviation, *RMSF* Root mean square fluctuation, *RG* Radius of gyration, *SASA* Solvent accessible surface area, *HB* Hydrogen bond

**Table 6:** The thioguanine with wild-type and its variants average simulation trajectories (RMSD, RMSF, RG, SASA, and HB)

Thioguanine (average protein trajectories)				
	Wild-type	Val18Ile	Arg139Cys	Arg139His
RMSD (nm)	0.415	0.35	0.314	0.446
RMSF (nm)	0.173	0.172	0.192	0.236
RG (nm)	1.502	1.491	1.506	1.574
SASA (nm^2^)	88.739	88.281	88.446	92.321
HB	96.947	97.852	98.071	93.392

*RMSD* Root mean square deviation, *RMSF* Root mean square fluctuation, *RG* Radius of gyration, *SASA* Solvent accessible surface area, *HB* Hydrogen bond

## Discussion

Leukemia is one of the most common cancers causing uncontrolled cell growth of leukocytes leading to mortality [32]. NUDT15 belongs to the NUDIX family and contains a hydrolase functional domain (position 9–145 amino acid) with a NUDIX box motif that metabolizes a variety of phosphorylated nucleosides such as oxidized nucleotide derivatives, capped mRNAs, diadenosine polyphosphates, and nucleoside triphosphate [33–35]. A recent study demonstrates the involvement of NUDT15 in the hydrolysis of cancer drugs such as azathioprine, mercaptopurine, and thioguanine [36]. Metabolism of these thiopurines has a significant impact on drug efficacy and side effects during the treatment of cancer [37]. Leucopenia is one of the common side effects that decrease the circulating white blood cells (granulocytes) due to drug metabolism [38]. Herein, we investigated the NUDT15 genetic polymorphisms contributing to change in the NUDT15 protein structure and its impact on azathioprine, mercaptopurine, and thioguanine metabolism by implementing a series of computational approaches: (1) mutation frequency assessment, (2) protein stability analysis, (3) molecular docking, and (4) dynamics simulation.

Firstly, we have assessed 83 missense variants of the NUDT15 gene across a variety of populations including East Asian, South Asian, European (Non-Finnish), European (Finnish), African/African American, Latino admixed American, and Ashkenazi Jewish. Among 83, three variants (Val18Ile, Arg139Cys, and Arg139His) were overrepresented in most of the population based on their allele frequency (Table 1). For instance, variants Val18Ile and Arg139Cys were more frequent in the East Asian population [39]. Likewise, Arg139His variant has been found dominant in Latino/Admixed American population from the GnomAD database. Additionally, these variants were prominently reported in patients with leukemia [40]. Thereby, it is essential to study the influence of these variants in the metabolism of cancer drugs. Next, we used structure stability tools [25–29] to study the protein stability acquired due to variants based on relative free energy (ΔΔG). The structure with the Val18Ile variant was predicted to be destabilizing by INPS-3D, CUPSAT, and SDM2 tools. Likewise, SDM2 and INPS-3D predicted that variant Arg139Cys may destabilize the NUDT15 structure. Similarly, Arg139His was predicted as a destabilizing mutation by CUPSAT and INPS-3D. These results suggest that structural rigidity is compromised in NUDT15 due to variants that might affect the protein-drug interaction. These destabilizing mutations greatly affect the stability and integrity of the structure that influences the protein function by altering the protein flexibility and binding efficiency [41].

Molecular docking was performed in order to discover the effect of NUDT15 variants on drug interaction. Notably, the least binding energy indicates a high affinity between the protein and ligand [42]. In our case, the higher binding affinity between NUDT15 and anti-cancer drugs would enhance the drug metabolism and decrease toxicity. The NUDT15 wild-type, Val18Ile, Arg139Cys, and Arg139His were docked with thiopurines (azathioprine, mercaptopurine, and thioguanine) and their binding energies were described in the results section (Table 3). Both hydrogen bond and van der Waals interaction play an active role in the protein-drug interaction (Fig. 1). According to our results (Table 3), azathioprine has the least binding energy with NUDT15 wild-type (− 7.6 kcal/mol) compared to mercaptopurine and thioguanine. Similar trends were noticed for NUDT15 variants with the binding energy for azathioprine with Val18Ile (− 7.3 kcal/mol), Arg139Cys (− 8.2 kcal/mol), and Arg139His (− 7.3 kcal/mol). These results suggest azathioprine may have the highest binding affinity with NUDT15 wild-type as well as with its variants that might effectively hydrolyze azathioprine to prevent leucopenia than other routinely recommended mercaptopurine and thioguanine [36, 43].

MD simulations were used to analyze the structural stability of the wild-type and its variants (Val18Ile, Arg139Cys, and Arg139His) with azathioprine, mercaptopurine, and thioguanine for 100 ns. Recently, MD simulations have been developed to understand the links between macromolecular structural conformation and function. Particularly, drug interaction with its target results in protein structural deviations, and conformational alterations, may also fluctuate the stability of the protein [44]. Our assessment of the MD trajectories, including RMSD, RMSF, RG, SASA, and HB (Tables 4, 5, and 6) demonstrates that NUDT15 and its variants were stable with azathioprine and mercaptopurine. Calculating RMSD trajectories provides information on structural deviation, conformational alteration, and stability [45]. The comparison of RMSD values showed minimal deviation for NUDT15 wild-type and variants with azathioprine and mercaptopurine, except Arg139His variant with all analyzed thiopurine drugs. The flexibility of the protein residue is represented by RMSF [46]. The residual fluctuations of NUDT15 structures with azathioprine, mercaptopurine, and thioguanine were established, which was optimal during the simulation demonstrating structural flexibility. RG is correlated with the volume of a protein’s tertiary structure and its overall conformational orientation [46, 47]. The RG trajectory analysis revealed a higher volume of a protein’s tertiary structure in Arg139His variant with thioguanine. Whereas, NUDT15 wild-type, Val18Ile, and Arg139Cys with azathioprine, mercaptopurine, and thioguanine were compactly packed throughout the simulation (Tables 4, 5, and 6). The protein’s external surface area that interacts with solvents is described by SASA [48]. Our SASA results suggest that the binding of azathioprine, mercaptopurine, and thioguanine with Arg139His structure caused significant changes in structural orientation, which indicates that solvent exposure with internal NUDT15 residues is higher. Also, HB analysis reveals the hydrogen bonding interaction between the protein and drug [49]. We found that the HB interaction of azathioprine and mercaptopurine has higher HB compared to thioguanine irrespective of any modifications in NUDT15 structures. Overall, by considering the outcome of docking and simulation analysis azathioprine was noticed with least binding energy than mercaptopurine and thioguanine as well as stable conformation structure in NUDT15 wild and its variants except Arg139His on azathioprine binding that may favor avoiding leucopenia.

## Conclusion

East Asian and Latin Admixed American populations are high-risk populations for the polymorphisms (Val18Ile, Arg139Cys, and Arg139His). All the variants were found to be destabilizing by mutation analysis. Azathioprine has higher binding interaction with NUDT15 wild-type and its variants compared to mercaptopurine and thioguanine according to docking analysis. Our simulation results suggest that azathioprine was stable with NUDT15 and its variants (Val18Ile and Arg139Cys), which might reduce the risk of leucopenia complications. Our study concludes that azathioprine will be a suitable drug for the treatment of leukemia.

### Supplementary Information

**Additional file 1: Figure S1.** Root mean square deviation of mercaptopurine with NUDT15 wild-type, V18I, R139C, and R139H. **Figure S2.** Root mean square fluctuation of mercaptopurine with NUDT15 wild-type, V18I, R139C, and R139H. **Figure S3.** Radius of gyration of mercaptopurine with NUDT15 wild-type, V18I, R139C, and R139H. **Figure S4.** Solvent accessible surface area of mercaptopurine with NUDT15 wild-type, V18I, R139C, and R139H. **Figure S5.** Hydrogen bonds of mercaptopurine with NUDT15 wild-type, V18I, R139C, and R139H. **Figure S6.** Root mean square deviation of thioguanine with NUDT15 wild-type, V18I, R139C, and R139H. **Figure S7.** Root mean square fluctuation of thioguanine with NUDT15 wild-type, V18I, R139C, and R139H. **Figure S8.** Radius of gyration of thioguanine with NUDT15 wild-type, V18I, R139C, and R139H. **Figure S9.** Solvent accessible surface area of thioguanine with NUDT15 wild-type, V18I, R139C, and R139H. **Figure S10.** Hydrogen bonds of thioguanine with NUDT15 wild-type, V18I, R139C, and R139H.

## Data Availability

We declare that most of the data generated are included in this study. Other data would be provided on reasonable request to corresponding author.
