# Supplementary material for: Comparative assessment of anti-cancer drugs against NUDT15 variants to prevent leucopenia side effect in leukemia patients
Source: J Genet Eng Biotechnol. 2023 Aug 9;21:82. doi: 10.1186/s43141-023-00538-1 (PMC10412517; doi:10.1186/s43141-023-00538-1)

**Figure S1** Root mean square deviation of mercaptopurine with NUDT15 wild-type, V18I, R139C, and R139H.


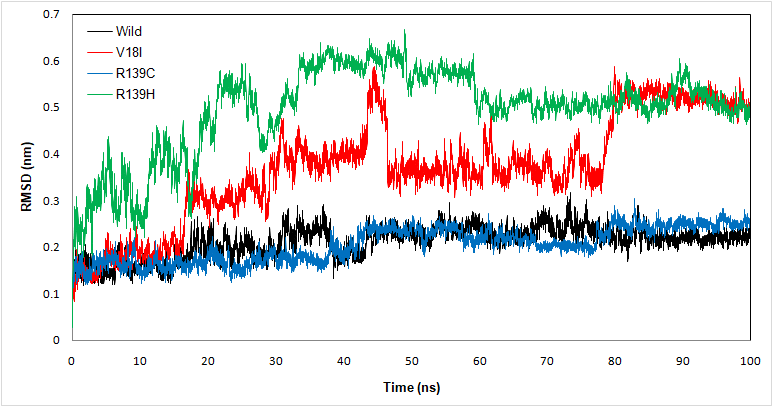


**Figure S2** Root mean square fluctuation of mercaptopurine with NUDT15 wild-type, V18I, R139C, and R139H.


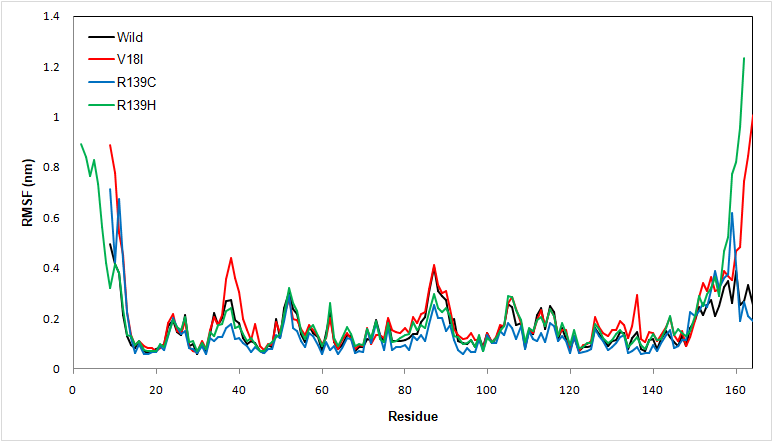


**Figure S3** Radius of gyration of mercaptopurine with NUDT15 wild-type, V18I, R139C, and R139H.


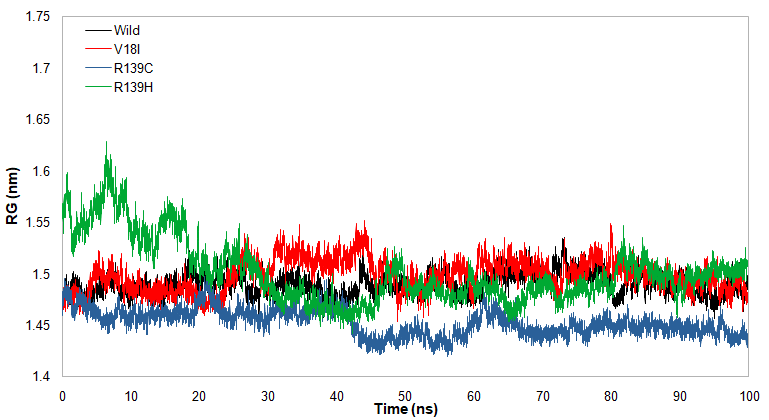


**Figure S4** Solvent accessible surface area of mercaptopurine with NUDT15 wild-type, V18I, R139C, and R139H.


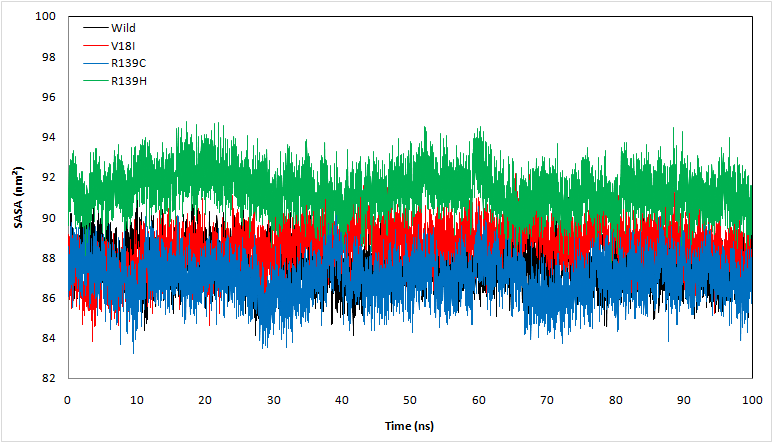


**Figure S5** Hydrogen bonds of mercaptopurine with NUDT15 wild-type, V18I, R139C, and R139H.


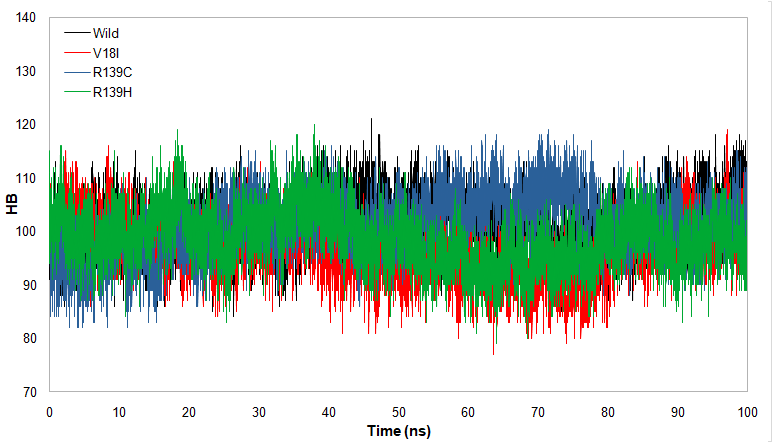


**Figure S6** Root mean square deviation of thioguanine with NUDT15 wild-type, V18I, R139C, and R139H.


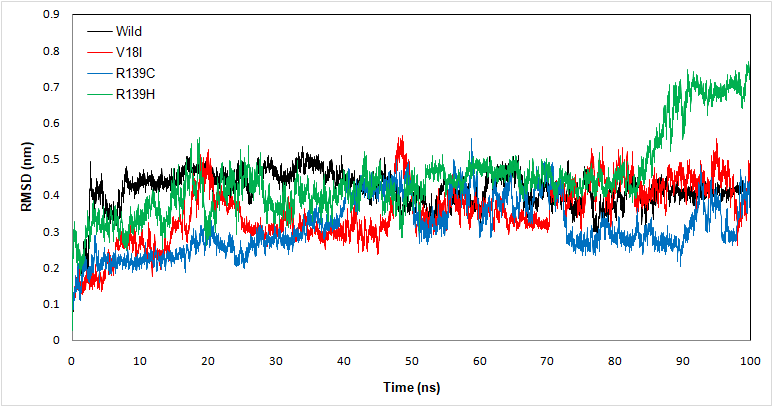


**Figure S7** Root mean square fluctuation of thioguanine with NUDT15 wild-type, V18I, R139C, and R139H.


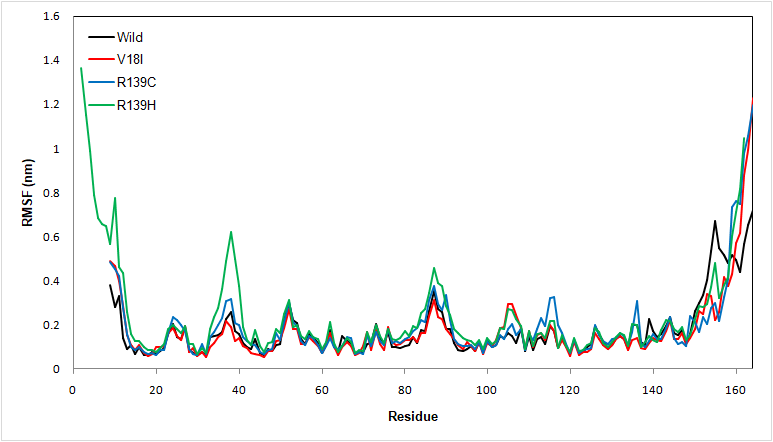


**Figure S8** Radius of gyration of thioguanine with NUDT15 wild-type, V18I, R139C, and R139H.


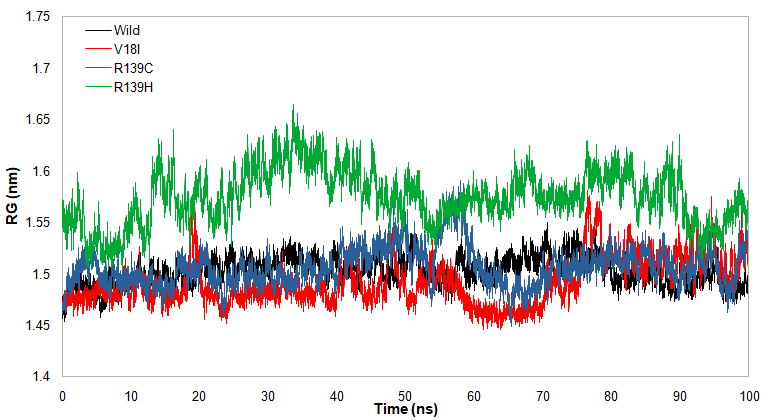


**Figure S9** Solvent accessible surface area of thioguanine with NUDT15 wild-type, V18I, R139C, and R139H.

**Figure S10** Hydrogen bonds of thioguanine with NUDT15 wild-type, V18I, R139C, and R139H.


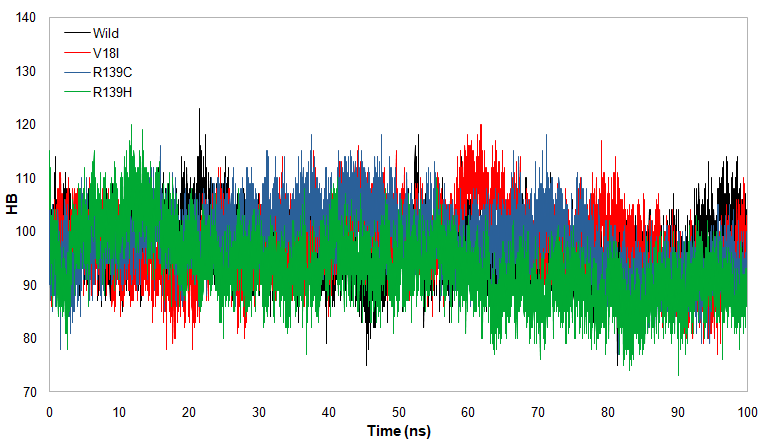

Supplement: Supplementary file 1 — Additional file 1: Figure S1. Root mean square deviation of mercaptopurine with NUDT15 wild-type, V18I, R139C, and R139H. Figure S2. Root mean square fluctuation of mercaptopurine with NUDT15 wild-type, V18I, R139C, and R139H. Figure S3. Radius of gyration of mercaptopurine with NUDT15 wild-type, V18I, R139C, and R139H. Figure S4. Solvent accessible surface area of mercaptopurine with NUDT15 wild-type, V18I, R139C, and R139H. Figure S5. Hydrogen bonds of mercaptopurine with NUDT15 wild-type, V18I, R139C, and R139H. Figure S6. Root mean square deviation of thioguanine with NUDT15 wild-type, V18I, R139C, and R139H. Figure S7. Root mean square fluctuation of thioguanine with NUDT15 wild-type, V18I, R139C, and R139H. Figure S8. Radius of gyration of thioguanine with NUDT15 wild-type, V18I, R139C, and R139H. Figure S9. Solvent accessible surface area of thioguanine with NUDT15 wild-type, V18I, R139C, and R139H. Figure S10. Hydrogen bonds of thioguanine with NUDT15 wild-type, V18I, R139C, and R139H. [file 43141_2023_538_MOESM1_ESM.docx]
